# Therapeutic Role of Mango Peels in Management of Dyslipidemia and Oxidative Stress in Obese Females

**DOI:** 10.1155/2021/3094571

**Published:** 2021-10-23

**Authors:** Farkhanda Arshad, Huma Umbreen, Iqra Aslam, Arruje Hameed, Kiran Aftab, Wahidah H. Al-Qahtani, Nighat Aslam, Razia Noreen

**Affiliations:** ^1^Department of Biochemistry, Government College University, Faisalabad-, Pakistan; ^2^Department of Nutritional Sciences, Government College University, Faisalabad-, Pakistan; ^3^Department of Biochemistry, University of Management and Technology, Sialkot Campus, Sialkot-, Pakistan; ^4^Department of Chemistry, Government College University, Faisalabad-, Pakistan; ^5^Department of Food Science and Nutrition, College of Food and Agriculture Sciences, King Saud University, Riyadh 11451, Saudi Arabia; ^6^Department of Biochemistry, Independent Medical College, Faisalabad-, Pakistan

## Abstract

Obesity is a chronic metabolic and noncommunicable disease that affects 50% of world population. Reactive oxygen species and oxidative stress are interconnected with the obesity and several metabolic disorders, gaining the attention of scientific community to combat this problem naturally. Among various fruits, mango as a yellow fruit is rich in polyphenols, carotenoids, terpenes, and flavonoids that act as antioxidants to protect against free radicals produced in the body. The present study was performed to explore *in vivo* antioxidant potential of mango peels against dyslipidemia and oxidative stress in overweight subjects. The female volunteers (*n* = 31) between 25 and 45 years of age having a body mass index (BMI) of 25.0-29.9 (overweight) were included in this study, while participants with complications as diabetes, hypertension, cardiovascular, and liver diseases were excluded. The treatment group consumed 1 g mango peel powder for 84 days. The subjects were analyzed for biochemical analysis, antioxidant status, and anthropometric measurements at baseline and end of the study period. Further, at the end of study, the safety evaluation tests were also performed. The results showed that upon consumption of mango peel powder, low-density lipoproteins (LDL), cholesterol, triglyceride, urea, and creatinine levels were decreased and high-density lipoprotein (HDL) level was increased (*P* ≤ 0.05), while thiobarbituric acid reactive substances (TBARS) showed increased antioxidant status (*P* ≤ 0.05) which suggests that mango peels have a strong management potential against oxidative stress and dyslipidemia in obese subjects.

## 1. Introduction

Obesity is a chronic disease characterized by accumulation of fat in adipose tissues resulting in increased body weight [1, 2]. It is considered a frequent metabolic disease, having 50% prevalence among adult masses throughout the world [3]. It is as common as is expected to be affecting 38% of adult women and 36.9% of adult men around the globe [4]. Obesity is the major causative agent for oxidative stress, which in return worsens the situation by changing the metabolite functions and stimulating the process of inflammation through cytokines [1, 5]. Furthermore, due to increased oxidative stress, innate immunity is triggered which gives rise to increased inflammation and lipid peroxidation that is ultimate route cause for different degenerative diseases [6, 7]. It is proposed that a cycle runs between obesity and oxidative stress which has threatening effects on cells and tissues, further depriving them of antioxidants [8]. Adipose tissues act as the gland which produces hormones, i.e., resistin, estrogen, and leptin as well as signaling protein such as cytokines. In response to increased adipocytes, unchecked adipokines are secreted which show increased immune response by producing reactive oxygen species (ROS) and free radicles, further enhancing the oxidative stress [9]. Moreover, oxidative stress damages pancreatic *β* cells and affects the production and release of insulin leading to altered transportation of glucose to the tissues, which may result in the development of metabolic syndrome [10]. Fruits and vegetables have biologically active compounds including carotenoids, anthocyanins, and polyphenols, which show prospective antioxidant action and can also strengthens defense against metabolic risk factors [11, 12]. Mango peel is an important part of the fruit considered inedible, rich in flavonoids, polyphenols, and carotenoids as antioxidants. It is about 15–20% of the fruit weight and is a major waste material of fruit processing sector [13]. Mango peel powders protect against fatty liver disease and impairment of renal and liver function as caused by medicines. Treatments of chronic diseases using the natural components as present in mango peels provide a better alternate of conventional medicines.

Keeping in view the potent health benefits of this by product, the present study was designed to probe its functional effect against most prevalent issue of society, i.e., dyslipidemia and oxidative stress related to obesity. Although there are few earlier research findings available showing the reduction in oxidative stress in rats, there are insufficient evidences available to address oxidative stress in human subjects using such an economical and natural resource.

## 2. Materials and Methods

### 2.1. Preparation of Raw Materials

Mangoes (*Mangifera indica*) variety Chaunsa were procured from the local market of Faisalabad, Pakistan, and washed and peeled. Afterwards, the separated peels were washed with warm water to deplete any sugars and dried at 60°C in a dehydrator (NESCO®/American Harvest). The dried peels were ground using a grinder (Panasonic MX AC 400 Mixer Grinder) and sieved to get even sized particles of 500–600 *μ*m size; then, the powder was stored in glass jars at 18°C and kept in the dry place [14].

### 2.2. HPLC Analysis

The mango peel extract was prepared from mango peel powder (MPP) according to the method followed by Tunchaiyaphum et al. [15]. The extract of MPP was prepared using 40% methanol in the ratio of 1 : 1, shaken gently for 5 minutes, and then added with 10 mL HCl (6 M). The prepared solution was kept for 2 hours in an oven at 50°C and filtered through microfilters of 0.2-0.4 microns, and the resulted sample was analyzed through gradient HPLC (Model LC-10; 32KARAT SOS, Shimazdu Japan) [16]. The mobile phase consisted of freshly prepared acetonitrile, dichloromethane, and methanol (ratio 60 : 20 : 20, respectively) and flow rate of sample (injection volume 15 *μ*L) was set at 0.8 mL/min [17].

### 2.3. Experimental Study Design

The nonprobability sampling technique (convenience sampling method) was used to select the sample. The participants were selected from Allied Hospital and Civil Hospital Faisalabad, Pakistan. The inclusion criteria was comprised of female subjects between 25 and 45 years of age having a BMI of 25.0-29.9 (overweight), while participants having any other complication as diabetes, hypertension, cardiovascular, and liver diseases were excluded. Out of a total 120 volunteers at the preliminary stage, 77 were excluded on the basis of diabetes and hypertension, and 12 were suffering from some sort of heart ailments. A total of 31 overweight subjects were divided in two groups: one treatment (21 subjects, taking selected doses of mango peels powder)and the other as control (10 subjects), which received no treatment but dietary guidelines were provided to both groups. All participants were requested to complete questionnaires about informed consent, physical examination, medical history, anthropometric measurements, and the food frequency questionnaire [18]. The dose was optimized and selected for all experiments and was provided in sachet (1 g MPP) that had to be taken twice a day half an hour before meal with one glass of water. The control group was advised to take one glass of water half an hour before meal for equivalence. High-fat and high-carbohydrate diets were restricted in all groups. The study was continued for 84 days after which the blood sample was taken again and BMI was calculated.

“Informed consent was obtained from all individual participants included in the study.”

### 2.4. Ethical Approval

The study was approved by the ethical review committee of the University for human and animal studies (ERC/GCUF/1966-IRB-566), and all studies were performed in accordance with the ethical standards of the 1964 Helsinki declaration and ethical principles of WHO (2008) and its later amendments or comparable ethical standards.

### 2.5. Blood Biochemistry and Antioxidant Analysis

The venous blood was processed to obtain plasma and serum under sterilized conditions at base line and at end of the study. A 4 mL blood was taken in the tube and let it stand for an hour to clot at room temperature. After clotting, centrifugation was performed for 10 minutes at 1000 rpm and serum containing aliquots were stored in a freezer at -4°C. For separation of plasma, blood was taken in EDTA tubes and kept at room temperature. After that, centrifugation was performed at 2000 rpm for 10 minutes and the supernatant (plasma) was separated and stored in a freezer at -4°C.

To measure complete blood count (CBC), the plasma was taken in a test tube and blood cells were analyzed through CBC autohaematology analyzer (NIPRO LE 1000 JAPAN). The serum was evaluated to find out the status of lipid profile using enzymatic colorimetric methods [19, 20]. Further, the serum was investigated for antioxidant status using thiobarbituric acid reactive substances (TBARS) by respective methods [21].

### 2.6. Safety Evaluation

For safety purpose, a discomfort questionnaire was filled up by the participants to report any disturbance after consumption of the MPP extract. Furthermore, to perform liver function test (LFT) and renal function test (RFT), colorimetric kit protocol [22] was adopted. The concentrations of bilirubin, urea, creatinine, etc. were determined through respective methods [23, 24].

### 2.7. Statistical Analysis

Analysis of variance (ANOVA) was used for data analysis [25] to know the significant difference. The least significant difference (LSD) was calculated to find the difference between means using SPSS (Version 17, USA). The results were declared to be significant at *P* ≤ 0.05.

## 3. Results and Discussion

### 3.1. HPLC Profiling

**Figure 1:**
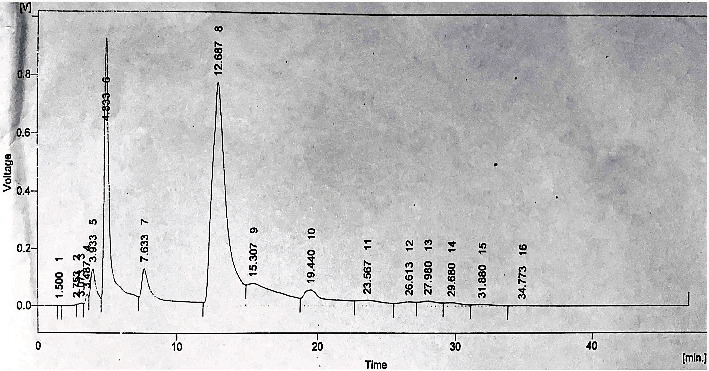
HPLC chromatogram of phenolic compounds of the mango peel extract. Peak identification of mango peel extract: 2, kaempherol; 3, quercetin; 6, gallic acid; 8, caffeic acid; 9, chlorogenic acid; 11, vitamin C; and 12, sinapicacid.

The gradient HPLC method was used for identification of polyphenolic compounds and antioxidants in the mango peel extract. The results of bioactive compounds showed a significant difference (*P* ≤ 0.05) among different parameters (Table 1). The mango peel extract showed the highest amount of caffeic acid while querecetin was observed to be the lowest. It was also observed that mango peel extract contained an effective amount of vitamin C (49.52 ppm) along with a significant amount of kaempherol, chlorogenic acid, and gallic acid. The HPLC chromatogram (Figure 1) showed peaks for retention times which were comparable with a known standard to identify the unknown phenolic compounds.

HPLC analyses have revealed that the methanolic extract of mango peel provided a higher concentration of phenolic compounds which is in accordance with other studies [26, 27]. The presence of major bioactive compounds, e.g., caffeic acid˃kaempherol˃gallic acid etc. in mango peels (Table 1) may not only lower the lipid level of plasma, cause inhibition of lipoprotein secretion, and result in removal of extra cholesterol from blood through bile acids but also are responsible in obesity reduction [28]. Yellow-colored fruits as orange, lemon, and mango are higher in flavonoids, polyphenols, terpenes, and carotenoids, which act as antioxidants to scavenge free radicals and reactive oxygen species [29, 30]. During this research, antioxidant concentration in the mango peel extract was found to be higher than the study conducted by Carvalho et al. [31]. The difference may be due to the varied climate situation, mango cultivars, and harvesting factors.

### 3.2. Anthropometric Data

**Figure 2:**
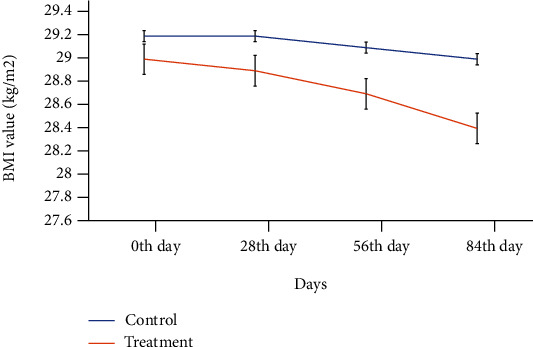
Effect of treatment days on the BMI value of obese subjects in the control and MPP-treated groups.

A total of 31 obese female volunteers (10 control and 21 treatment subjects) were between the ages of 25 and 45 belonging to nearly similar BMIs (Table 2). The results showed that the BMI value decreased nonsignificantly with time (0^th^ -84^th^ day) in both groups. However, this reduction was more pronounced (from 29 to 28.4 kg/m^2^) in case of subjects in a group treated with mango peel extract (Figure 2).

The mango peel extract decreased weight gain in the treatment group compared to the control group. Previous studies have proved that due to consumption of mango peel, the body weight decreases and the antioxidant level increases [32]. Moreover, a nonsignificant reduction in BMI for treatment as compared to the control group at the end of experimental period may be attributed to the combined effect of bioactive compounds responsible for weight management including caffeic acid and kaempherol [33, 34].

### 3.3. Efficacy Study

All the subjects were analyzed for blood biochemistry. The results showed that hemoglobin, eosinophils, ESR (erythrocyte sedimentation rate), monocytes, polymorphs, and TLC (total leukocyte count) did not change significantly (*P* ≤ 0.05) among the control and treatment groups, while lymphocytes were found to be increased significantly in the MPP-treated group compared to the control group (Table 3).

Moreover, the data showed a significant difference (*P* ≤ 0.05) for the lipid profile test among the control and treatment groups of obese subjects (Table 3). After consumption of MPP, significant reduction in triglyceride (-4.63%), TC (-13.12%), and LDL (-9.04%), while 9.97% increase in the HDL level in the treatment group compared to the control group were observed.

**Figure 3:**
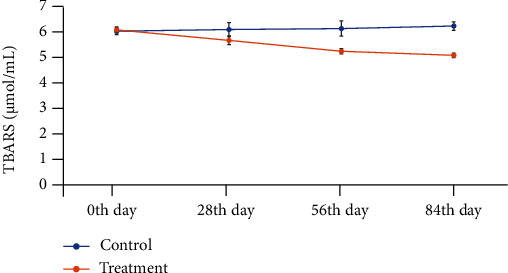
Reduction in the TBARS (*μ*mol/mL ±SEM) value with increase in treatment days.

There was a significant difference (*P* ≤ 0.05) between the groups for thiobarbituric acid reactive substance (TBARS) values (Figure 3). The antioxidant status in the treatment group was significantly increased after the intake of mango peel powder (MPP) due to decrease in the TBARS value with increasing number of treatment days (0^th^-84^th^).

The biochemical profile showed that lymphocytes were increased in the concentration while count of other blood cells was decreased after intervention. In a review conducted by Moler and Soft [35], it was described that antioxidants affect the blood cell count over a longer period of time; therefore, during a short time as in the case of the present study, the results are not much pronounced.

Furthermore, the results showed the management of dyslipidemia with reduced plasma triglyceride and cholesterol levels in obese subjects treated with mango peel powder. Thus, it showed protective effects against vascular damage caused by oxidation of LDL. These findings are in accordance with those found by Duttaroy and Jørgensen [36]. The reduction in the concentration of LDL cholesterol may be accredited to bioactive compounds with higher antioxidant potential present in mango peels. They cause downregulation of proteins involved in lipogenesis; therefore, lipogenesis is repressed, whereas energy utilization is enhanced that give positive correlation to the adiposity. In the present study, HDL concentration was increased in the treatment group consuming MPP, which is important in the transport of extra cholesterol from cells and tissues to the liver for ultimate degradation. These findings are in accordance with the research work of Muruganandan et al. [37], which also showed an increase in the concentration of HDL in diabetic rats. With treatment of MPP, the antioxidant status was improved in subjects and TBARS values were decreased. TBARS are byproducts of lipid peroxidation and comprise of aldehydes and lipid hydroperoxides which are enhanced during oxidative stress. It describes lipid peroxidation in terms of MDA and is indicative of antioxidant status. Therefore, the consumption of mango peel powder not only controls the lipid level but also is effective against lipid peroxidation [38].

### 3.4. Safety Evaluation

Both groups were also evaluated for safety test including discomfort questionnaire to find out any adverse effect on the subjects. The discomfort questionnaire showed no significant disturbance except reduction in appetite in the treatment group as compared to the control group. For liver function test (LFT) and renal function test (RFT), serum bilirubin, alkaline phosphate (ALP), alanine aminotransferase (ALT), aspartate aminotransferase (AST), blood urea, and creatinine, respectively, showed significant difference at *P* ≤ 0.05 (Table 4).

Bilirubin concentration is an indicator of liver and bile disorders. Moreover, bilirubin results in decreased blood cholesterol concentration through increased HDL and decreased LDL levels. The present study showed an increased bilirubin concentration in the treatment group that may be due to an increased antioxidant status of the patient and reduced inflammatory condition [39]. Furthermore, the consumption of MPP reduced alkaline phosphatase (ALP) concentration and had required effects on adipocytes and fatty deposits. The ALP isozyme was reported to be expressed in adipocytes which ultimately raise the fat deposits during the enhanced adipogenesis in obesity [40]. As the ALP level is an indicator of obesity so reduction of the ALP level by MPP showed safety of mango peel doses to reduce the fatty deposits. The degenerative changes in the liver and heart cause the increase concentration of ALT and AST which are used as biomarker for diagnostic purpose of liver damage [41]. Moreover, the nonalcoholic fatty liver diseases (NAFLD) are most common metabolic syndrome related to obesity in which the increased level of hepatic enzymes leads towards the sever obesity. The ALT and AST are released into the blood circulation in response to the cell deterioration process due to high peroxidation of lipids, and in this condition, the antioxidant level of the body is much decreased. This increase in enzyme concentrations is due to the high-fat diet during the intervention period so mango peel powder serves to reduce these enzyme concentrations [42]. However, mango peel supplementation is also reported to improve the concentration of these enzymes in treated obese subjects due to higher polyphenolic content with appreciable antioxidant power [43]. In the current research, urea and creatinine were decreased in the treatment group compared to the control group and showed the efficiency of MPP against any deterioration in kidney function. Creatinine is actually a metabolic product of muscle which is transported to the kidney through renal glomeruli for filtration and some dispose in urine. Creatinine concentration increases with age, gender, and BMI [44], and these results are in accordance with the results demonstrated by Morsi et al. [45].

## 4. Conclusion

Obesity is increasing at an alarming rate which becomes further dangerous due to production of reactive oxygen species (ROS). Based upon the present research results, it can be concluded that bioactive compounds from agricultural waste (mango peels) present a comprehensive remedy to fight against the ROS produced in the body. Thus, these are helpful in preventing damages caused, using economical resources that otherwise go waste and become a source of pollution.

## Figures and Tables

**Table 1:** HPLC profile of mango peel extract.

Compounds	Retention time (Rt)	Amount (ppm)	Area (mv·S)	Area (%)
Kaempherol	2.35 ± 0.02^g^	187.7 ± 0.04^b^	2813.37 ± 3.4^d^	93.5 ± 1.2^a^
Quercetin	3.07 ± 0.01^f^	11.87 ± 0.01^f^	224.82 ± 1.2^g^	0.2 ± 0.2^g^
Gallic acid	4.83 ± 0.01^e^	95.90 ± 0.02^c^	26641.69 ± 3.5^b^	22.2 ± 0.1^c^
Caffeic acid	12.68 ± 0.03^d^	247.39 ± 0.59^a^	53782.26 ± 3.3^a^	44.8 ± 3.5^b^
Chlorogenic acid	15.30 ± 0.04^a^	95.52 ± 0.04^c^	12250.82 ± 2.7^c^	10.2 ± 0.09^d^
Vitamin C	23.56 ± 0.1^b^	49.52 ± 0.04^d^	2476.19 ± 1.3^e^	2.1 ± 0.03^e^
Sinapic acid	26.61 ± 0.7^a^	14.76 ± 0.04^e^	1136.40 ± 1.4^f^	0.9 ± 0.01^f^

^a–g^Different letters in columns show a significant difference at *P* ≤ 0.05.

**Table 2:** Average baseline anthropometric measurement of volunteers.

Parameters	Groups		Probability values^∗^
	Control (*n* = 10)	Treatment (*n* = 21)	
Age (years)	33.7 ± 1.31	34.1 ± 0.92	0.15
BMI (kg/m^2^)	29.2 ± 0.46	29.0 ± 0.46	0.12

Data is presented as the means ± SE of control and treatment group. ^∗^Probability values (*P ≤0.05*) show significant differences between the groups.

**Table 3:** Blood biochemistry of the control and treatment groups of obese subjects.

Parameters	Groups		Probability values^∗^
	Control (*n* = 10)	Treatment (*n* = 21)	
Complete blood count (g/dL)			
Hemoglobin	12.44 ± 0.94	12.38 ± 0.84	0.96
Eosinophils	0.14 ± 0.024	0.14 ± 0.024	1
ESR	17.80 ± 0.80	16.60 ± 0.67	0.28
Lymphocytes	36.60 ± 0.40^b^	39.20 ± 0.91^a^	0.03
Monocytes	0.24 ± 0.024	0.24 ± 0.024	1
Polymorphs	60 ± 0.04	54.60 ± 2.46	0.59
TLC	7720 ± 185.47	7660.0 ± 60.0	0.76
Lipid profile (mg/dL)			
Triglycerides	146.80 ± 1.73^a^	140.30 ± 2.48^b^	0.02
Total cholesterol	188.90 ± 3.06^a^	164.10 ± 3.35^b^	0.01
HDL	46.10 ± 1.73^b^	50.70 ± 1.35^a^	0.05
LDL	134.90 ± 3.51^a^	122.70 ± 2.55^b^	0.01
TBARS (*μ*mol)	6.08 ± 0.07^a^	5.66 ± 0.05^b^	0.001

Data are presented as the means ± SEM of the control and treatment groups. ^∗^Probability values (*P* ≤ 0.05) show significant differences between the groups. ^a,b^Different letters in rows show a significant difference at *P* ≤ 0.05. TC: total cholesterol; HDL: high-density lipoprotein; LDL: low-density lipoprotein; Hb: hemoglobin; ESR: erythrocyte sedimentation rate; TLC: total leukocyte count.

**Table 4:** Safety analysis for the control and treatment groups of obese subjects.

Parameters	Groups		Probability values^∗^
	Control (*n* = 10)	Treatment (*n* = 21)	
Liver function test			
Bilirubin direct (mg/dL)	0.40 ± 0.05	0.41 ± 0.05	0.90
Bilirubin indirect (mg/dL)	0.23 ± 0.012^b^	0.38 ± 0.05^a^	0.03
Bilirubin total (mg/dL)	0.64 ± 0.048	0.48 ± 0.15	0.35
ALP (U/L)	244.0 ± 12.88^a^	188.40 ± 0.40^b^	0.002
ALT (U/L)	53.200 ± 3.30	44.00 ± 3.67	0.09
AST (U/L)	39.00 ± 2.19^a^	30.40 ± 0.40^b^	0.004
Renal function test (mg/dL)			
Blood urea	39.60 ± 1.50^a^	34.6 ± 0.74^b^	0.02
Creatinine	0.80 ± 0.06^a^	0.74 ± 0.05^b^	0.02

^∗^Probability values *P* ≤ 0.05 show significant differences between the groups. Different letters (ab) in the column show significant difference at *P* ≤ 0.05. Data is presented as the means ± SEM of the control and treatment groups. ALP: Alkaline phosphatase; ALT: alanine aminotransferase; AST: aspartate aminotransferase.

## Data Availability

All types of data are included in the research manuscript.
